# Structure Function Studies of Photosystem II Using X-Ray Free Electron Lasers

**DOI:** 10.1146/annurev-biophys-071723-102519

**Published:** 2024-07

**Authors:** Junko Yano, Jan Kern, Vittal K. Yachandra

**Affiliations:** Molecular Biophysics and Integrated Bioimaging Division, Lawrence Berkeley National Laboratory, Berkeley, California, USA

**Keywords:** manganese, oxygen-evolving complex, photosystem II, photosynthetic water oxidation, X-ray crystallography, X-ray free electron laser

## Abstract

The structure and mechanism of the water-oxidation chemistry that occurs in photosystem II have been subjects of great interest. The advent of X-ray free electron lasers allowed the determination of structures of the stable intermediate states and of steps in the transitions between these intermediate states, bringing a new perspective to this field. The room-temperature structures collected as the photosynthetic water oxidation reaction proceeds in real time have provided important novel insights into the structural changes and the mechanism of the water oxidation reaction. The time-resolved measurements have also given us a view of how this reaction—which involves multielectron, multiproton processes—is facilitated by the interaction of the ligands and the protein residues in the oxygen-evolving complex. These structures have also provided a picture of the dynamics occurring in the channels within photosystem II that are involved in the transport of the substrate water to the catalytic center and protons to the bulk.

## 1. INTRODUCTION

**Figure 1:**
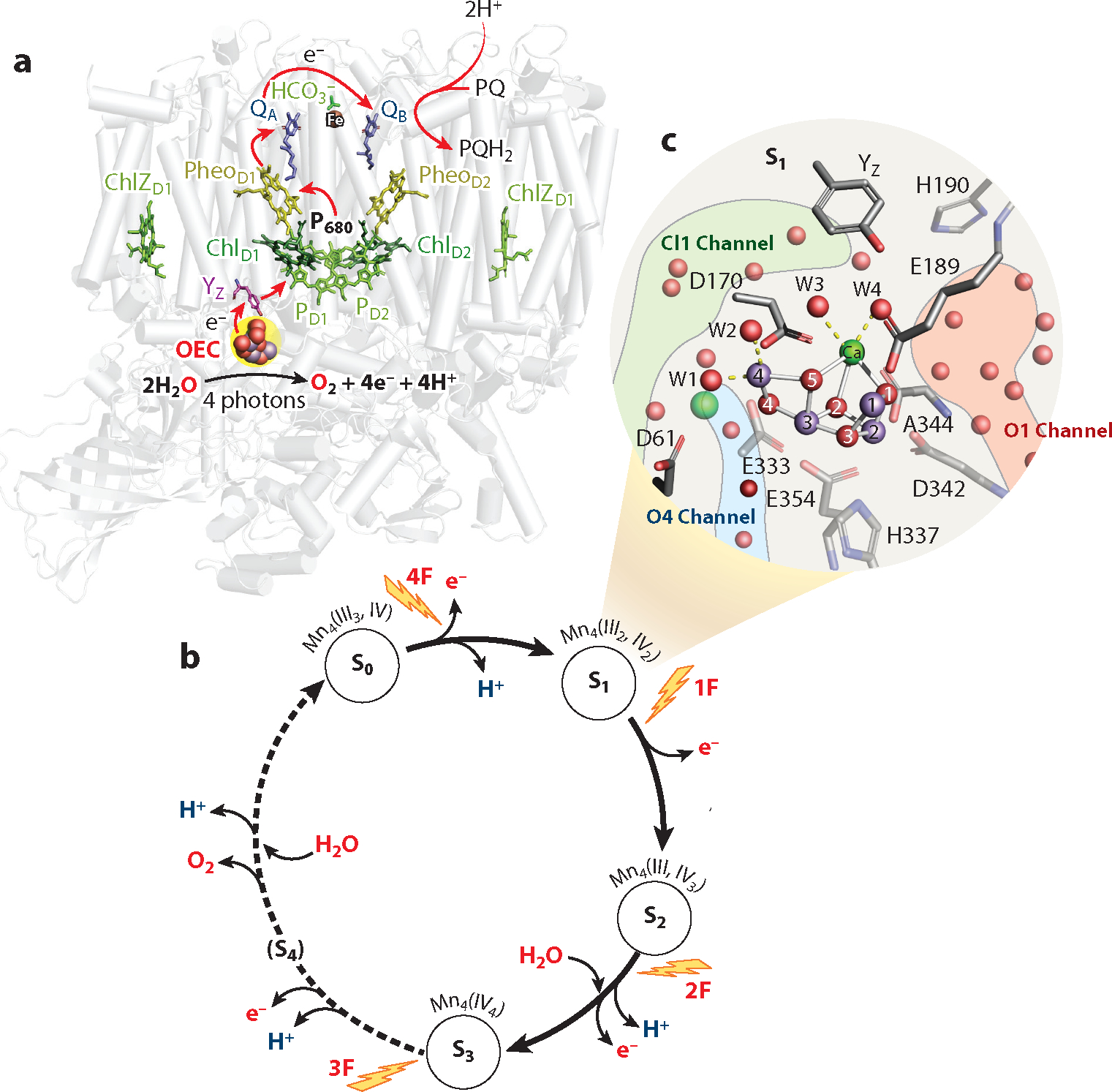
Photosystem II (PS II) structure and the oxygen-evolving complex. (*a*) The structure of PS II, with the protein backbone shown in gray. The groups involved in charge separation and transfer across the membrane lipid bilayer section of the thylakoid membrane are shown in color. The red arrows show the electron transfer path from the pair of primary donor chlorophylls, P_D1_ and P_D2_, via Chl_D1_ and Pheo_D1_ to the acceptor quinone molecules Q_A_ and Q_B_, respectively. The oxygen-evolving complex (OEC) with the Mn_4_CaO_5_ cluster is located in the boundary region between the membrane and the extrinsic peptides, with the redox-active tyrosine Y_Z_ located between the complex and the primary donor. (*b*) The S-state Kok clock shows the kinetic model for water oxidation, with the intermediate S_0_ to S_4_ states and the respective oxidation states of Mn. Absorption of a photon advances each S-state to the next with the release of protons and the introduction of waters as shown. (*c*) The structure of the OEC in the native dark-adapted PS II with the ligands to the Mn and Ca shown (Mn in *purple*, Ca in *green*, and O in *red*). Free waters are shown as red spheres, and the starting points of the O1, O4, and Cl1 channels, which connect the OEC to the bulk, are indicated in light red, light blue, and light green, respectively. Figure adapted from Reference 6 (CC BY 4.0).

Most oxygen, on which life as it exists today on earth depends, is generated by plants, algae, and cyanobacteria via the light-induced oxidation of water in photosystem II (PS II), a membrane-bound pigment−protein complex embedded in the thylakoid membrane of all oxygenic photosynthetic organisms (Figure 1a). This reaction is one of the most important, life-sustaining chemical processes occurring on such a large scale in the biosphere. The water-oxidation reaction probably first appeared in nature approximately 2.5 to 3 billion years ago in the precursors to present-day cyanobacteria, although the exact timeline or lineage is the subject of much debate (7, 121, 130, 143). An essential component in the evolution of oxygenic photosynthesis is the Mn_4_CaO_5_ catalytic complex, which can store four oxidizing equivalents, a prerequisite to catalyze the four-electron oxidation of two water molecules to dioxygen with low overpotential. In addition to the four electrons, four protons are released during the reaction. The resulting membrane potential and proton gradient across the membrane drive the synthesis of ATP molecules. The electrons are utilized for the generation of NADPH, and both ATP and NADPH are used to drive the reduction of CO_2_ to carbohydrates via the Calvin cycle. The electrons and protons produced during the water-oxidation reaction in PS II are thus ultimately used to store energy; this is nature’s elegant way of converting the energy from sunlight into chemical energy. It is this form of energy-rich compounds created by photosynthesis, as well as the byproduct of O_2_, on which all higher life on earth depends.

The Mn_4_CaO_5_ metal center in PS II, referred to as the oxygen-evolving complex (OEC), catalyzes the four-electron and four-proton redox chemistry of water and consists of an oxo-bridged cluster of four Mn and one Ca, with several ligands to both the Mn and Ca provided by the surrounding amino acid residues (Figure 1a). The four-electron redox reaction is coupled with the one-electron photochemistry occurring at the PS II reaction center, where the primary charge separation by the absorption of sunlight occurs, and subsequent electron transfer takes place through several vectorially arranged pigment cofactor molecules (124, 145).

The structure of the reaction center of PS II is similar to the photosynthetic bacterial reaction centers (37), but in contrast to them, it also includes the OEC. PS II is found as a dimer with a molecular weight of approximately 700 kDa in all known oxygenic photosynthetic organisms, and the two monomers are related by a twofold axis perpendicular to the membrane plane. Each monomer (Figure 1a) consists of 17 or 18 membrane integral subunits with a total of 35–36 transmembrane helices and 3–4 peripheral subunits (65, 124, 141, 161). The exact composition of the smaller peripheral peptides that are both intrinsic and extrinsic to the membrane differs among cyanobacteria, algae, and higher plants. The monomer in cyanobacteria is characterized by a pseudo-twofold symmetry, with the D1, CP47, and PsbI subunits related to the D2, CP43, and PsbX subunits, respectively, via the pseudo-symmetry axis. Each monomer contains 35 chlorophyll a (Chl) molecules, 11–12 all-*trans* β-carotene molecules, 1 OEC (Mn_4_CaO_5_ cluster), 1 heme b, 1 heme c, 2 or 3 plastoquinones, 2 pheophytins, 1 nonheme Fe, and approximately 20–25 lipids.

Figure 1a shows the electron transfer chain in the structure of the PS II monomer, where the initial light harvesting, primary charge separation, charge stabilization, and electron transfer take place on the picosecond-to-microsecond timescale (113, 124). The P_680_ reaction center in the D1 and D2 subunits is the primary electron donor that traps the light energy delivered from the inner antenna subunits (CP43 and CP47 subunits) or the outer antenna complexes of PS II (LHC II in plants and the phycobilisome in cyanobacteria). The exact location of primary charge separation, e.g., whether the subsequent acceptors Chl_D1_ and pheophytin (Pheo_D1_) are also involved in this step, was a matter of debate for many years (89, 117). Recent experiments (see 158) clearly showed that the excited state of the primary donor P_680_∗ rapidly (within approximately 3 ps) leads to the initial charge-separated state between Chl_D1_ and Pheo_D1_, and eventually to the acceptor, plastoquinone Q_A_ (in approximately 250 ps). The last electron transfer step to the final electron acceptor plastoquinone Q_B_ takes place on the timescale of hundreds of microseconds (36), stabilizing the charge-separated state. Plastoquinone Q_B_ leaves the pocket on the acceptor side of PS II after accepting two electrons and two protons as QH_2(B)_, plastoquinol, and is released into the membrane matrix for transfer to the cytb_6_f complex, which mediates electron transfer between PS II and photosystem I (88). On the donor side of PS II, the photo-oxidized cationic radical P680^•^+ is reduced by a tyrosine residue, Y_Z_ (D1-Tyr161), to generate a neutral tyrosine radical, Y_Z_^•^, which oxidizes the Mn_4_CaO_5_ complex and initiates the water oxidation process at the catalytic metal center of the OEC. Y_Z_ has a strong H-bonding interaction with the nearby D1-His190. This close interaction is essential for modulating the protonation state of Y_Z_, and it was suggested to exist even before structural details were available showing that Y_Z_ plays an important role in the proton-coupled electron transfer processes at the OEC during water oxidation (138).

Inspired by the period-four oscillation in flash-induced oxygen evolution of PS II discovered by Joliot and colleagues (68) in 1969, Kok and colleagues (79) proposed a five-state kinetic model for photosynthetic oxygen evolution, known as Kok’s S-state cycle. The model comprises four intermediates (S_0_, S_1_, S_2_, and S_3_) and one transient S_4_ state, which precedes dioxygen formation from two water-derived oxygens bound at the Mn_4_CaO_5_ cluster in the OEC. Thus, the Mn_4_CaO_5_ cluster in the OEC couples the four-electron oxidation of water with the one-electron photochemistry occurring at the PS II reaction center by acting as the locus of charge accumulation and the center where the water-oxidation chemistry, including O–O bond formation and dioxygen release, occurs.

During one cycle of the catalytic reaction, the OEC consumes two water molecules; one is introduced into the cycle during the S_2_→S_3_ transition and the second during the S_3_→[S_4_]→S_0_ transition. Meanwhile, in addition to four electrons, four protons are released from the catalytic reaction in the pattern of 1:0:1:2 for the S-state transitions S_0_→S_1_→S_2_→S_3_→S_0_ (122, 136) (although some differences are observed when populating the high-spin S_2_ state; 11). All four S-states of PS II can be populated by illumination of dark-adapted PS II (as it relaxes to the S_1_ state in the dark) with 0, 1, 2, or 3 flashes.

During the four-step reaction, the protein residues not only modulate the redox potentials of the Mn_4_CaO_5_ cluster, but also provide pathways for electrons, protons, substrate H_2_O, and product O_2_. PS II orchestrates the well-controlled catalytic reaction close to the thermodynamic potential (113). This redox leveling achieved by storing redox equivalents on the metal cluster allows the system to perform all steps in the water oxidation reaction, with the driving force of approximately 1.1 V provided by the Y_Z_/Y_Z_^+^ couple in each of the transitions (113). Moreover, this multistep oxidation proceeds without the release of damaging intermediates of the water-oxidation process, such as hydroxide radicals, peroxide, or superoxide, that can be detrimental to the protein and to the chemistry occurring at the OEC (118).

In this review, we focus on the current understanding of the water-oxidation reaction in PS II, centered around the Mn_4_CaO_5_ cluster, with emphasis on recent X-ray free electron laser (XFEL)-based room-temperature structures of intermediates. We also discuss the importance of the interplay between the cofactors and their environment for enabling multielectron and multiproton redox chemistry (128).

## 2. GEOMETRIC AND ELECTRONIC STRUCTURE OF THE Mn_4_CaO_5_ CLUSTER IN THE S_1_ (DARK, RESTING) STATE

The mechanism of photosynthetic water oxidation and the structure of the catalytically active site have been subjects of intense study by many groups with a wide range of methods ever since Mn was identified as an essential element for this reaction in algae and moss by Pirson (108) in 1937. Electron paramagnetic resonance (EPR) spectroscopy (29, 87), infrared (IR) (4, 100–102), mass spectroscopy (16, 35, 60, 125), biochemical (5, 22, 70, 99, 103), and theoretical (53, 67, 82, 85, 119, 127, 132) studies, and especially early X-ray diffraction (XRD) (43, 55, 69, 86, 141, 161) using synchrotron radiation at cryogenic temperatures and X-ray spectroscopy [Extended X-Ray Absorption Fine Structure (EXAFS), X-Ray Absorption Near Edge (XANES), and X-Ray Emission Spectroscopy (XES)] (32, 106, 149, 156, 157), have provided valuable insights into the structure of the Mn_4_CaO_5_ cluster and the mechanism of water oxidation in PS II.

The detection in the 1980s of the light-induced formation of an EPR signal [commonly referred to as the multiline signal (MLS)] associated with the S_2_ state was an important step; the MLS had a very rich, hyperfine structure, which is the signature of a mixed-valence Mn cluster containing both Mn^III^ and Mn^IV^ oxidation states (39–41). The MLS and its many variants, as well as the EPR signals that have been discovered from the other S-states (namely the S_0_, S_1_, and S_3_ states), have been used for modeling the possible structure and the valence state of the Mn_4_CaO_5_ cluster through the Kok cycle (1, 2, 9, 14, 17, 38, 59, 92, 160). Structural studies of the Mn_4_CaO_5_ complex were initially mostly based on EXAFS studies. These were critical in determining the presence of di- and mono-μ-oxo-bridged Mn-Mn at approximately 2.7 and 3.3 Å, respectively (33, 34, 47, 90, 94, 107, 146–148), and Mn oxo-bridged to Ca at approximately 3.4 Å (23–25, 83, 111, 150), as well as the metal–ligand distances. These distances showed little change during the S_1_→S_2_ transition (90, 148), but more clear changes were observed during the S_2_→S_3_ transition (52, 84) and in the S_0_ state (51, 114, 115). EXAFS experiments were also the first to show that the structure contained a unique Ca, possibly oxo-bridged to all four Mn (23–25, 83, 111, 150), and polarized EXAFS using crystals of PS II was the first to show that the Mn_4_CaO_5_ structure was an open-cubane-like structure (153),although the exact damage-free (152), three-dimensional structure was only determined later by crystallographic studies using XFELs at cryogenic or room temperature.

Information about oxidation states, the electronic structure, and how charge and spin densities around the Mn_4_Ca cluster change as it progresses through the catalytic cycle is also important for understanding the mechanism of water-oxidation chemistry. EPR (29, 56), IR (101, 102), and X-Ray Absorption Spectroscopy (XAS) and XES (31, 32, 49, 57, 58, 93, 116, 155) studies have shown that the Mn_4_Ca cluster changes in its formal oxidation states from (III_3_,IV), to (III_2_,IV_2_), to (III,IV_3_), to (IV_4_) in the S_0_→S_1_, S_1_→S_2_, and S_2_→S_3_ transitions. Although there is overall consensus that the Mn cluster is oxidized as it proceeds through the S_0_ to S_3_ states, the exact chemical state and the degree of delocalization of the charge and spins are not yet clear (48, 49). It is important to note that the distribution of spin and charge may be important not only for PS II, but also for other catalytic reactions involving transition metal compounds (129). This is especially the case if these reactions have to proceed via very high-valence species or if storage of species with very high oxidation or reduction potentials during the reaction is necessary (54).

### 2.1. X-Ray Crystallography of Photosystem II at Cryogenic Temperatures Using Synchrotron Radiation

Early X-ray crystallography studies at 3.0–3.8-Å resolution using synchrotron radiation sources were successful in determining the overall structure of PS II (43, 55, 69, 86, 161), but the information they provided regarding the geometry and ligand environment of the Mn_4_Ca cluster was limited, as the crystal diffraction data suffered from the progressive reduction of the native high-valence manganese cluster back to the Mn^II^ state by the X-ray beam, which was accompanied by a disruption of the Mn_4_Ca oxo-bridged structure (152). Therefore, EXAFS, which can use a very low dose of X-rays, was required to provide a set of possible structures in the relaxed S_1_ state and in the other higher oxidation states. This situation changed when Umena and coworkers (141) determined the structure of PS II at 1.9 Å using a low X-ray dose, limiting the damage to only approximately 25% of Mn atoms being reduced by X-rays. However, there were intrinsic limitations to the use of X-rays from synchrotron sources, such as the requirement to freeze the crystals because of radiation damage issues.

### 2.2. X-Ray Crystallography of Photosystem II at Room Temperature Using X-Ray Free Electron Lasers

With the advent of XFEL sources, beginning with the Linac Coherent Light Source at Stanford about a decade ago, that generate femtosecond X-ray pulses, it became possible to outrun X-ray-induced damage and collect data at room temperature. In general, XFEL experiments rely on the so-called diffract-before-destroy approach, where the probed sample volume is completely destroyed by the individual XFEL pulse and replaced by a fresh sample volume before the arrival of the next XFEL shot (15, 18, 98, 131). However, as the information (diffraction pattern) is collected within the femtosecond pulse length, slower X-ray damage processes, which usually happen on the picosecond timescale, do not affect the obtained data. Therefore, one can expose the studied sample to a very high X-ray dose, and cryogenic conditions are not necessary. This capability opened up the possibility of collecting structures and spectroscopic data of PS II in all of the S-states in a time-resolved manner under physiological conditions (71–74).

More recently, the structures of all of the S-states have been determined at approximately 2.0-Å resolution at room temperature (63, 64, 73, 133, 135, 159). In addition, several structures for time points during the transitions between the S-states, including the S_3_→S_0_ transition where the O–O bond is formed, were obtained (6).

In this review, we focus on recent XFEL-based structural studies at room temperature and the changes that they observed in the catalytic center and the channels that connect the center to the bulk, providing conduits for protons and water that are required for the water-oxidation reaction.

## 3. INTERMEDIATE S-STATE STRUCTURES

**Figure 2:**
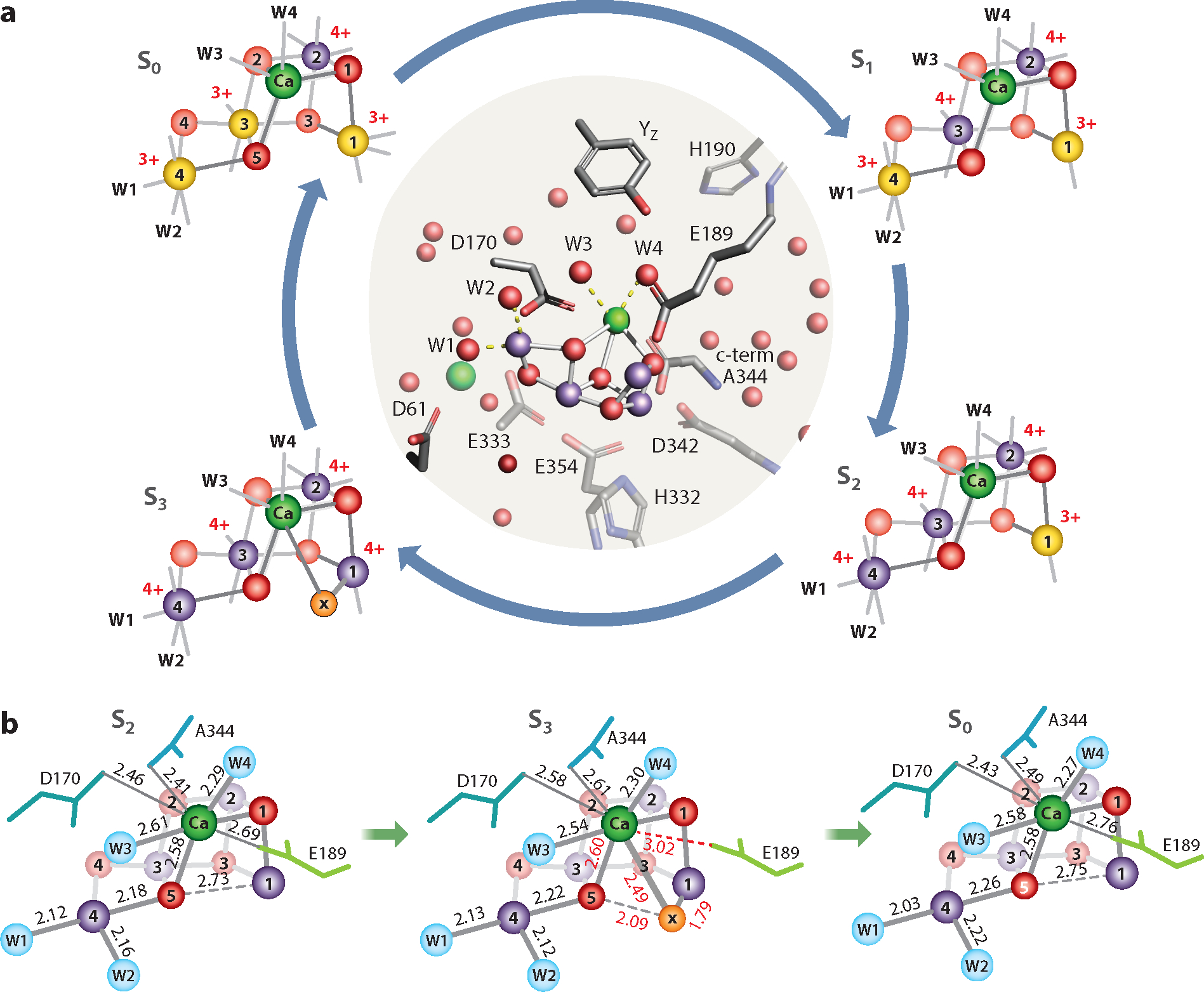
Changes in the structure of the Mn_4_Ca cluster during the S-state cycle. (*a*) The center panel shows the ligand environment of the oxygen-evolving complex (OEC). The right-open, cubane-like structure is preserved throughout the S-state cycle. The Mn atoms are shown in yellow (oxidation state 3+) and purple (4+). Ca atoms are shown in green, and O atoms are shown in red. The most reduced state is Mn_3_^III^Mn^IV^ in S_0_, and the most oxidized state is Mn_4_^IV^ in S_3_. O_X_, which is introduced during the S_2_→S_3_ transition and is bridged to Mn1 and Ca, is shown in orange. (*b*) The structural changes among S_2_, S_3_, and S_0_ are shown, with the distances between atoms given in angstroms. The distance between O5 and the newly introduced O_X_ is 2.09 Å. The movement of E189 away from Ca upon the S2→S3 transition is indicated by the increase of the Ca–O distance from 2.69 Å to 3.02 Å, which is again shortened in the S_0_ state when O_X_ is no longer present. Figure adapted from Reference 73.

The introduction of XFELs has enabled us to investigate the subtle differences of the intermediate S-state structures by overcoming the issue of radiation-induced changes, which is particularly a problem for redox-active metal clusters like the OEC in PS II (75). Figure 2 shows the structures of the S-state intermediates of the cluster, with the oxidation states of Mn and changes in the ligand environment indicated (73).

### 3.1. The S_1_ State

The structure of the Mn_4_CaO_5_ cluster in the dark resting S_1_ state is shown in Figure 1a. The cluster is in a right-open structure with no bond between Mn1 and O_5_; the Mn_4_–O5 distance is approximately 2.2 Å, whereas the Mn1–O5 distance is approximately 2.7 Å, clearly too long for a bond between Mn1 and O5 to exist. Thus, Mn1 is pentacoordinate in the S_1_ state. The formal oxidation state of the four Mn atoms is Mn^III^_2_Mn^IV^_2_, and therefore, this state is EPR silent. Mn1 and Mn4 are considered as Mn^3^+ in the S_1_ state (80, 105, 127, 151). The metal ligation sphere is completed by four waters (W1–W4), two bound to Mn4 and two to Ca, as well as six carboxylate ligands (each ligating two metals) and one histidine ligand.

The main open question connected to the structure of the S_1_ state is the protonation state of the bridging and terminal oxygen ligands, which cannot be deduced from the current data. For example, the protonation state of W2, which has been theorized to be either OH or OH_2_, has consequences for the possible deprotonation mechanism in the later stage of the reaction cycle (28).

### 3.2. The S_2_ State

Upon the S_1_→S_2_ transition (first flash), one Mn is oxidized from +3 to +4 (Figure 2). The fundamental geometry of the cluster in the S_2_ state, a right-open structure that has been shown by crystallography and EXAFS studies, remains unchanged (73, 153, 154). One important open question regarding the S_2_ state is what the functional roles of the high-spin and low-spin isomers observed in this state are. The low-spin form, with spin *S*_total_ = 1/2 configuration (the MLS discussed above), in which Mn4 is +4, is considered to be consistent with the form observed in the crystal structure, with a geometry similar to the S_1_ state. In addition, EPR studies show multiple signals at higher g-values assigned to the high-spin forms, and their population depends on the species, sample preparation (pH), and presence of additives (8–10, 12, 13, 109). The debate centers around the structural nature of the high-spin form(s); whether it is a right-closed (or left-open) structure; and whether it could mimic the transient state during the S_2_→S_3_ transition, a possibility that has been suggested based on theoretical studies (26, 27, 104). This structural isomorphism related to the high- and low-spin isomers in the S_2_ state has not been supported by cryogenic EXAFS structures (19, 20) or room-temperature XFEL-based structures (64, 73) of the S_2_ state.

Notably, the biggest difference between the S_1_ and S_2_ state structure is found in the secondary coordination environment; a water (W20) that is located at the entrance of the O4 channel of the OEC near the bridging oxygen O4 disappears during the S_1_→S_2_ transition. This implies that the hydrogen bonding network is changed between the S_1_ and S_2_ states due to the charge density changes of the cluster. While the oxidation of Mn in this transition is not accompanied by the release of a proton from the OEC to the bulk (122, 136), this observation suggests that there is likely a motion of protons that influences the hydrogen bonding network around the OEC.

### 3.3. The S_3_ State

In the S_3_ structure, the last open coordination site of Mn1, which is pentacoordinate and in oxidation state 3+ in the S_2_ state, is filled with an oxygen [O_X_ (73) or O6 (134)]. It is likely a hydroxo ligand, as proposed by some theoretical studies and indicated by simulations of the EPR data that satisfy the *S*_total_ = 3 spin state configuration of the S_3_ state (21, 30). The O_X_ ligand forms a bridge between Mn1 and Ca. To accommodate the ligation by this additional oxygen, the Ca coordination environment changes; Glu189, ligated to Mn1 and Ca in the S_2_ state, moves away from Ca to become a monodentate ligand with Mn1 (Figure 2b), and O_X_ bridges Ca and Mn1. The coordination number of Ca remains the same, at eight. The observed elongation of the Mn1–Mn4 and Mn1–Mn3 distances by approximately 0.2 and 0.07 Å, respectively, relative to the S_2_ state is caused by the incorporation of O_X_ into the cluster. This change in coordination of Mn1 from 5- to 6-coordinate is in line with the proposed oxidation of Mn1 from +3 to +4 in the S_2_→S_3_ transition (21, 32, 64).

The close proximity of O_X_ and O5 indicates that this could be an O–O bond formation site in the next transition, as has been suggested in different theoretical models in the past (see, for example, 127, 151). Other studies suggested instead that O_X_ and O5 already form a bond (an oxo-oxyl/peroxo/superoxo bond) in the S_3_ state (110, 134, 135). For the peroxo and superoxo bond formations between O_X_ and O5, one and two, respectively, of the Mn atoms would need to be reduced to 3+. However, based on the observation that the distance between O_X_ and O5 is approximately 2.05 Å (64, 73), and that Mn is oxidized in S_3_ to be all 4+—as shown by EPR (21), XAS (32), and XES (64, 123) studies—we do not expect the O–O bond formation to occur at this stage.

### 3.4. The S_0_ State

The third laser flash advances the S_3_ state to the most reduced S_0_ state. In this transition, first, the water oxidation is completed and O_2_ is released; subsequently or simultaneously, binding of one new water molecule occurs, resetting the catalytic cycle. The room-temperature crystal structure of the S_0_ state shows the loss of O_X_ and the return of the cluster structure to a motif similar to the dark-stable S_1_ state. In parallel, the ligation of D1-Glu189 to Ca is reestablished (Figure 2b), and W20—which disappeared during the S_1_→S_2_ transition—reappears in the S_0_ state. The oxidation state of S_0_ is Mn_3_^III^Mn^IV^, as shown by EPR and X-ray spectroscopy analysis (32, 46, 91, 92, 115). The most probable assignment of the oxidation state of each Mn for all states is shown in Figure 2a.

## 4. STRUCTURAL SEQUENCE OF EVENTS DURING THE S-STATE TRANSITION

**Figure 3:**
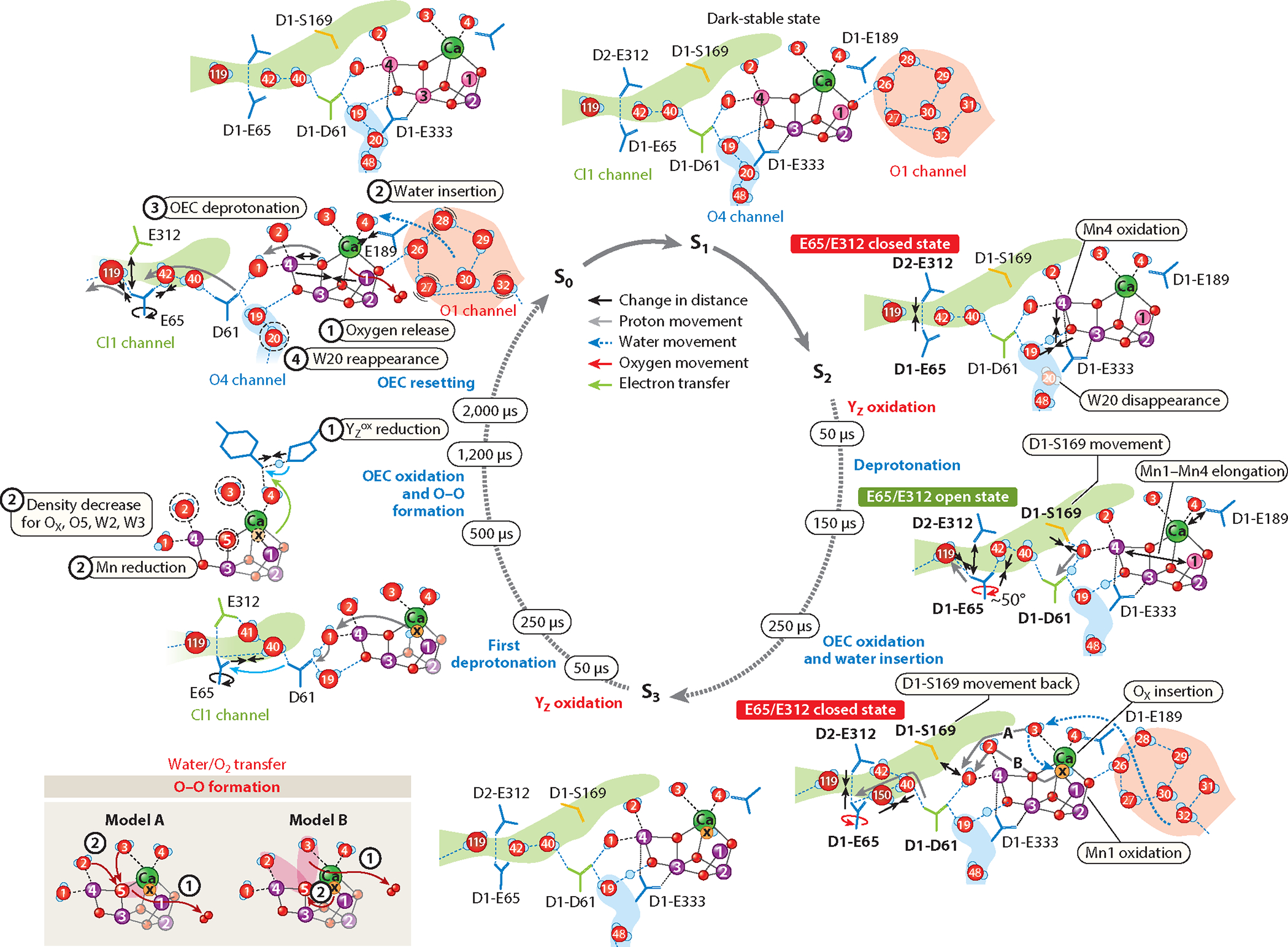
Mechanistic proposals for water oxidation in photosystem II (PS II). The circle in the center shows the S-states and the time sequence. The S_1_ state structure, including ligands and surrounding waters, is shown at the top; the oxidation states of Mn are Mn_2_^III^ (*purple*) and Mn_2_^IV^ (*magenta*). Upon absorption of a photon, Mn4 is oxidized from III to IV, with the only major change being the disappearance of water W20. Transitioning to S_3_ involves oxidation of Mn1, along with elongation of the Mn1–Mn4 distance and the insertion of O_X_ between Mn1 and Ca. Delivery of water to the cluster via the water wheel and Ca ligands is shown by the blue dashed arrow. Changes are seen in the proton gate area formed by residues Glu65 and Glu312 of the Cl1 channel. During the S_3_→S_0_ transition, there is a decrease in electron density at O_X_, O5, W3, and W4, with O_X_ eventually disappearing upon formation of the S_0_ state and two deprotonation events occurring, as shown by the closing and opening of the gate at D1-Glu65. Two likely O–O bond formation mechanisms are shown in the left panel. Figure adapted from References 6 (CC BY 4.0) and 63 (CC BY 4.0). Additional abbreviation: OEC, oxygen-evolving complex.

Figure 3 summarizes the structural changes at and around the donor side of PS II during the catalytic cycle, obtained from room-temperature crystallography studies. In addition to the steady-state structures captured at room temperature, the snapshots of the structures during the S_2_→S_3_ and S_3_→S_0_ transitions show the sequence and the progression of each of the events.

### 4.1. S_2_ to S_3_

The S_2_→S_3_ transition, initiated by the second laser flash, is a critical step, as it is coupled with the first water binding to the Mn_4_CaO_5_ cluster and the release of one proton. In the S_2_→S_3_ transition, the earliest event (<50 μs) observed in the time-resolved study is the motion of Y_Z_, together with the D1-His190 and D1-Asn298 residues, and the motion of D1-Glu189, which is located next to Ca. These changes can be explained by the transfer of an electron from Y_Z_ (Y_Z_ → Y_Z_^•+^) to P680^•+^. The oxidation of Y_Z_ is expected to change the H-bonding network along the residues D1-Y_Z_-His190-Asn298 and the surrounding waters in their vicinity. The formation of the positive charge at the Y_Z_–His190 pair could trigger the shift of Glu189 away from Ca that is observed in the crystal structure at 50 μs after the second flash. In the next step (<150 μs), the elongation of the Mn1–Mn4 distance becomes visible, together with the appearance of the additional ligand O_X_ (64, 73) or O6 (135) as a bridging ligand between Ca and Mn1. Concomitantly measured time-resolved XES data indicate that the oxidation of Mn1 from 3+ to 4+ is directly coupled to the insertion of this ligand (64).

### 4.2. S_3_ to S_0_

In the S_3_→S_0_ transition, one of the earliest events is, again, the oxidation of the redox active Y_Z_, indicated by a motion of Tyr161 and His190, observed by 50 μs after the third flash (Y_Z_^ox^ formation). Y_Z_ then returns to the reduced state after receiving an electron from the OEC through the last oxidation of the cluster. Several spectroscopic studies show the presence of several phases in the S_3_→S_0_ transition (50, 57, 112). These include some processes prior to the oxidation of the OEC and reduction of Y_Z_^ox^ back to Y_Z_. IR and photothermal beam deflection spectroscopic data suggest that, during this lag phase, the first proton is released (78, 102). In the crystal structure, this likely corresponds to the time period of 200–500 μs, in which no major changes are observed in the Mn_4_O_6_Ca cluster.

In the early stage of the 500 to 1,200 μs period, the last oxidation event (transient S_4_ state formation, with Mn^IV^_4_O^•^ or Mn^IV^_3_Mn^V^) occurs; subsequently, the reduction of Mn takes place. The O–O bond formation should be triggered by this final oxidation event of the OEC, and the distance change observed at Y_Z_–D1-His190 in the 500–730 μs time period suggests that the reduction of Y_Z_^ox^ takes place through the electron transfer from the OEC to Y_Z_.

The transient formation of the S_4_ state triggers the O–O bond formation, and the release of molecular oxygen occurs, accompanied by the four-electron reduction of the cluster, a return of one substrate water, and the release of a second proton. The four-electron reduction may proceed in one step with the O–O bond formation and immediate release of O_2_ or in two steps with the presence of an intermediate before the release of molecular oxygen from the OEC. In the latter case, a peroxo species formed by an initial two-electron reduction appears to be the most likely intermediate. The crystallography data show that there is a delay between the onset of O–O bond formation (500–730 μs), as indicated by the Y_Z_–D1-His190 distance or rotation, and the decrease of the O_X_ electron density and, therefore, the onset of the O_2_ release, as evidenced also by the Mn1–Mn4 distance contraction observed after 730 μs. The delay indicates the presence of an intermediate state, possibly a peroxide-like species, which would support a mechanism of two consecutive two-electron reduction steps for the water oxidation reaction.

## 5. CHANNELS

**Figure 4:**
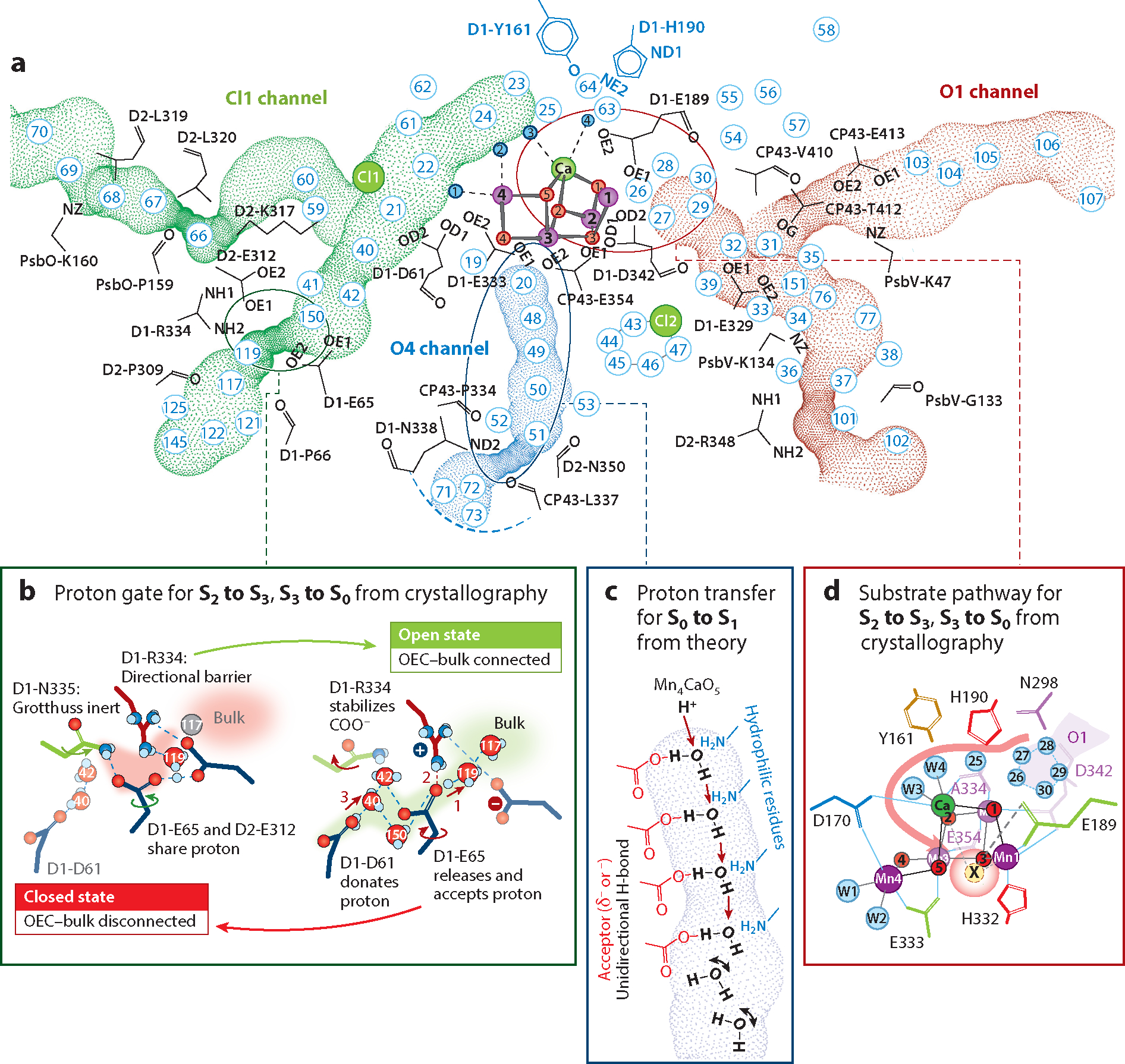
Proton and water substrate channels in photosystem II (PS II). (*a*) The O1, O4, and Cl1 channels connecting the oxygen-evolving complex (OEC) to the lumen are shown in red, blue, and green, respectively, with some lining residues indicated. The numbers indicate crystallographically identified waters in the channels. The circles indicate areas expanded in panels *b*, *c*, and *d*. (*b*) The proposed proton gate in the Cl1 channel, with green shading showing the open gate between the OEC and the bulk and red showing the gate closed around the D1-Glu65, D2-Glu312, and D1-Arg334 residues. (*c*) The unidirectional H-bond sequence from O4 in the Mn_4_CaO_5_ cluster to the bulk. There is a discontinuity or bottleneck region between W51 and W72, making it unlikely that the O4 channel acts as a water channel, as suggested by Sakashita et al. (120). (*d*) The water wheel in the O1 channel consisting of five waters (W26–30) that exhibit high mobility between the S-states and are postulated to transport water from the bulk to the Mn_4_CaO_5_ cluster via the Ca atom ligands W3 and W4. Panels *a*, *b*, and *d* adapted from Reference 63 (CC BY 4.0).

During the catalysis, the spatially controlled transport of the substrate water, electrons, and protons between the catalytic center and the inside of the thylakoids (lumen) is essential for catalytic efficiency. The electron transfer along a chain of cofactors is described in Section 1 and Figure 1. It should be noted that proton-coupled electron transfer is thought to be essential to allow efficient coupling between the OEC and the electron transfer chain and that a well-designed hydrogen bond network around the OEC seems to be a prerequisite for the charge leveling that allows subsequent oxidization of the OEC by the same oxidant (Y_Z_) in each of the S-states (see, e.g., 78, 97, 113, 124, 126, 138). In this section, we mainly discuss substrate water and proton transport. There have been several theoretical and experimental studies (44, 45, 61, 66, 81, 95, 142, 144) identifying the channels that facilitate substrate intake and proton release in PS II. The three channels that are thought to be important for water and proton transport are the O1 channel (large channel), the O4 channel (narrow channel), and the Cl1 channel (broad channel) (Figure 4). All three channels start near the OEC and extend outward toward the lumenal side of the complex to the bulk water. These three channels are observed in various oxygenic photosynthetic organisms, with some species-specific variations, despite the fact that these species have been separated for a long time in the evolutionary landscape (62). This signifies that the channels play a crucial role in the survival of these organisms that is related to the water-splitting reaction.

### 5.1. Substrate Channel

During the water-oxidation reaction, two substrate waters are consumed at the OEC. It is known that one binds to the OEC during the S_2_→S_3_ transition, and the other binds during the S_3_→S_0_ transition, right after the release of O_2_. Theoretical studies have followed the access of bulk water to the catalytic site based on the earlier crystallography data (44, 45, 61, 66, 81, 96, 141, 144). The room-temperature crystallography data taken during the S_2_→S_3_ and S_3_→S_0_ transitions showed the high mobility (higher crystallography B-factor and root mean square deviation of positions at different time points) of waters in the O1 channel compared to other channels, leading to the hypothesis that the O1 channel serves as a substrate channel from the lumenal side of the bulk water (6, 63, 64). The pentacluster of waters in the vicinity of the OEC, known as the water wheel region (Figure 4d), is considered to be a point at which substrate waters can enter into the OEC.

Several studies have proposed that Ca-bound water (W3) may serve as the entrance point for substrate water (3, 76, 139) in the S_2_→S_3_ transition. The simultaneously collected crystallography and X-ray emission data suggest that the substrate water insertion to the last open coordination site of Mn (Mn1) occurs concomitantly with the oxidation of this Mn (3+ to 4+) (64). W3 could then be filled by water that arrives from the water wheel region of the O1 channel via W4 to W3. In this scenario, the Ca ligand environment serves as a water gate of the OEC.

The S_3_→S_0_ transition also involves the insertion of one substrate water into the OEC. Once O_2_ is released, refilling of the cluster with a new substrate water needs to occur. There is no clear evidence of the disappearance of a second oxygen density in the cluster, besides O_X_. Therefore, the data suggest that the O_2_ release and water refilling are highly coordinated, and refilling likely occurs via a terminal water already ligated to the OEC. Extensive motion of water molecules around the water wheel region is also observed in the S_3_→S_0_ transition, and therefore, it was proposed that PS II uses the O1 channel for the substrate intake in both the S_2_→S_3_ and S_3_→S_0_ transitions via the Ca ligand environment (6, 64).

### 5.2. Proton Channel

In the water-oxidation reaction, four protons are released from the OEC, with a release pattern of 1:0:1:2 for the S_0_→S_1_, S_1_→S_2_, S_2_→S_3_, and S_3_→S_0_ transitions. The mobility of waters is much lower in the Cl1 or O4 channels, in contrast to the O1 channel, as evidenced by B-factor analysis of the room-temperature crystal structures (63, 64). These channels form a tighter hydrogen bonding network than the O1 channel, which may be necessary for efficient control of proton release.

In the S_0_→S_1_ transition, one proton is released in parallel to the one-electron oxidation of Mn. It has been proposed that O5 is protonated in the S_0_ state; if this is the case, this proton is a likely candidate for release during this transition. To date, no experimental study has followed the time-resolved structural changes of the cluster or the channels for this transition. However, theoretical studies by Takaoka et al. (137) and Sakashita et al. (120) suggest that the O4 channel could be the proton channel in this transition. This conclusion is based on the donor-to-acceptor direction of the hydrogen bonding network from the bulk to the OEC formed in the S_1_ state (a post-proton-transfer orientation) (Figure 4c). Such a donor-to-acceptor orientation is also supported by the single crystalline molecular dynamics simulation that is based on the S_1_-state room-temperature crystallography data (42). A very fast proton transfer has also been detected via time-resolved IR spectroscopy, supporting the use of the tight H-bond network of the O4 channel for proton transfer in this transition (126).

There is consensus that the S_1_→S_2_ transition is not accompanied by a proton release from the OEC to the bulk, and only electron transfer occurs (oxidation of Mn). However, a delocalization of proton(s) within the OEC may still occur to compensate for the accumulation of an additional charge at the cluster.

The S_2_→S_3_ transition involves the substrate binding to the Mn_4_CaO_5_ cluster, forming a Mn_4_CaO_6_ cluster. Recent crystallography data suggest that O_X_, which is likely a hydroxo ligand, originates from the Ca-ligated water, W3, a conclusion that is in line with other experimental data (76, 140). This transfer process would be accompanied by a release of one proton. The Cl1 channel appears to be the most probable pathway for proton release during this transition in both theory (42) and experiment (62, 63). In the steady-state structure, the Cl1 channel has a bottleneck region formed by residues D1-Glu65, D2-Glu312, and D1-Arg334, which blocks the transfer of water or protons (Figure 4b). However, the crystallography data show that the motion of this bottleneck region is reversible around the time that the O_X_ density becomes visible at the OEC; a rotation of D1-Glu65 by approximately 50° is seen in the time-resolved data at 150 μs after the second flash, which leads to a rearrangement of the H-bond network. This residue then reverts back to its steady-state configuration by 250 μs after the second flash.

Interestingly, similar changes in the bottleneck area are observed during the S_3_→S_0_ transition as well, but these changes occur twice: first around 500 μs and again around 1,200 μs. Some spectroscopic studies have suggested that a deprotonation event occurs prior to the last oxidation of the OEC (77, 78), and the earlier motion of the Cl1 bottleneck region may correspond to this event. The later motion of this region coincides with the onset of the O_X_ disappearance at the OEC. It is likely related to the recovery of the OEC, which involves the insertion of a substrate water after the release of O_2_. Thus, we hypothesize that the Glu65–Glu312–Arg334 region serves as a proton gate that controls the shuttling of the proton from the OEC to the bulk and minimizes back-reactions. The gate can exhibit two forms—a closed state that does not allow proton egress and an open state for efficient proton transfer to the bulk (63) (Figure 4b). The conformational changes at this gate region are closely intertwined with the electronic changes at the cluster with Mn oxidation and O_X_ insertion. Finally, the amino acid coordination environment, the hydrogen-bonding network around the OEC, and the waters in the channels reset to those of the S_0_ state configuration. This process includes the recovery of W20 in the O4 channel, and this channel is proposed to be involved in proton release during the S_0_→S_1_ transition.

## 6. MECHANISTIC UNDERSTANDING OF THE WATER OXIDATION REACTION

The PS II crystal structures taken throughout the Kok cycle revealed details of the molecular processes for photosynthetic water oxidation. The delay between the O_X_ density changes (onset of approximately 500 μs) and the disappearance of O_X_ (onset of 1,200 μs) suggest that there is an intermediate, such as a bound peroxide, that lives for a certain period. Several O–O bond formation sites have been proposed in the literature (53, 85, 119, 127, 132, 151). Among these, the O5–O_X_ site is in best agreement with the structural data because of the proximity of the two oxygen atoms and the reduced occupancy of O5 observed at approximately 1,200 μs; however, two other possibilities in which O5 reacts with either W2 or W3 and O_X_ replaces O5 cannot be dismissed at this point (Figure 3, inset).

The O–O bond formation is triggered by the last oxidation event (transient S_4_ state formation, with Mn^IV^_4_O^•^ or Mn^IV^_3_^V^), and subsequently, the reduction of Mn takes place. The Y_Z_–D1-His190 distance change is visible in the structure between 500 and 730 μs after the third flash, suggesting that the reduction of Y_Z_^ox^ takes place during this time, through the electron transfer from the OEC to Y_Z_. At the cluster, the evidence of the highest oxidized S_4_ state has not been observed in the crystal structure. A possible interpretation for this absence of observations would be that this state only transiently exists, and the O–O bond formation happens immediately (i.e., forming an intermediate that is reduced by two electrons from the S_4_ state).

The structural studies provide experimental support for a two-step reduction mechanism of the Mn_4_CaO_5_-O_X_ cluster upon the O–O bond formation and O_2_ release with a transient intermediate, most likely a bound peroxide. Moreover, a series of changes are spread over various locations of the protein and start right after the third flash, close to the redox-active Y_Z_ area, and extend up to several milliseconds that are needed for rearranging the hydrogen bonding and water network around the OEC. In particular, the recovery process of the environment that surrounds the OEC after the release of O_2_ seems to take time. In contrast, the replacement of substrate oxygen after the release of O_2_ seems to be instantaneous, given that we do not observe a state in which a second oxygen besides O_X_ disappears.

## 7. PERSPECTIVE AND OUTLOOK

Elucidating the mechanism for the O–O bond formation in PS II has been a quest ever since the discovery that Mn was required for oxygen evolution in plants and algae by Pirson (108) in 1937 and the observation of the period-four oscillation in flash-induced oxygen evolution by Joliot and colleagues (68) and Kok and colleagues (79) in the 1960s and 1970s. Owing to the knowledge gained particularly in the last two decades, a mechanistic understanding of the water-oxidation reaction in nature is coming within reach. The high-resolution crystal structure of the PS II resting S_1_ state by Umena et al. (141) under cryogenic temperature conditions took us closer to envisioning where the potential O–O bond formation site could be. The advancement of techniques, especially XFELs, in the past decade has led to further breakthroughs in our understanding of how PS II cycles during the catalysis.

The main results of the studies described in the above sections are (*a*) the catalytic reaction at the metal center is coordinated by the extended area of the amino acid and water network; (*b*) the sequence of proton, electron, substrate, and product transport during the four-electron and four-proton water-oxidation reaction is highly coordinated; and (*c*) the delicate charge balancing observed is most likely essential for the reaction to proceed with minimal overpotential and without the generation of dangerous or wasteful side products.

More broadly, this case study of the water-oxidation reaction sets the stage for future investigations into the fundamental principles of light-driven enzymatic multielectron and multiproton reactions, which are enabled through the interplay among the metal center, the protein environment, and the water network.
